# Efficacy of R_2_CHA_2_DS_2_-VA score for predicting thromboembolism in Thai patients with non-valvular atrial fibrillation

**DOI:** 10.1186/s12872-021-02370-2

**Published:** 2021-11-12

**Authors:** Komsing Methavigul, Poom Sairat, Rungroj Krittayaphong, for the COOL-AF Investigators

**Affiliations:** 1 Department of Cardiology, Central Chest Institute of Thailand, Nonthaburi, Thailand; 2 Division of Cardiology, Department of Medicine, Faculty of Medicine Siriraj Hospital, Mahidol University, 2 Wanglang Road, Bangkoknoi, Bangkok, 10700 Thailand

**Keywords:** R_2_CHA_2_DS_2_-VA, CHA_2_DS_2_-VASc, Thromboembolism, Non-valvular atrial fibrillation, NVAF, Anticoagulant

## Abstract

**Background:**

There is no data specific to the addition of renal dysfunction and age 50–64 years as risk parameters to the CHA_2_DS_2_-VA score, which is known as the R_2_CHA_2_DS_2_-VA score, among NVAF patients. Accordingly, the aim of this study was to validate the R_2_CHA_2_DS_2_-VA score for predicting thromboembolism in Thai NVAF patients.

**Methods:**

Thai NVAF patients were prospectively enrolled in a nationwide multicenter registry from 27 hospitals during 2014–2020. Each component of the CHA_2_DS_2_-VA and R_2_CHA_2_DS_2_-VA scores was scored and recorded. The main outcomes were thromboembolism, including ischemic stroke, transient ischemic attack (TIA), and/or systemic embolism. The annual incidence rate of thromboembolism among patients in each R_2_CHA_2_DS_2_-VA and CHA_2_DS_2_-VA risk score category is shown as hazard ratio (HR) and 95% confidence interval (95% CI). The performance of the R_2_CHA_2_DS_2_-VA and CHA_2_DS_2_-VA scores was demonstrated using c-statistics. Net reclassification index was calculated. Calibration plat was used to assess agreement between observed probabilities and predicted probabilities of both scoring system.

**Results:**

A total of 3402 patients were enrolled during 2014–2020. The average age of patients was 67.38 ± 11.27 years. Of those, 46.9% had renal disease, 30.7% had a history of heart failure, and 17.1% had previous stroke or TIA. The average R_2_CHA_2_DS_2_-VA and CHA_2_DS_2_-VA scores were 3.92 ± 1.92 and 2.98 ± 1.43, respectively. Annual thromboembolic risk increased with incremental increase in R_2_CHA_2_DS_2_-VA and CHA_2_DS_2_-VA scores. Oral anticoagulants had benefit in stroke prevention in NVAF patients with an R_2_CHA_2_DS_2_-VA score of 2 or more (adjusted HR: 0.630, 95% CI 0.413–0.962, *p* = 0.032). The c-statistics were 0.630 (95% CI 0.61–0.65) and 0.627 (95% CI 0.61–0.64), for R_2_CHA_2_DS_2_-VA and CHA2DS2-VA scores respectively. NRI was 2.2%. The slope and R2 of the calibration plot were 0.73 and 0.905 for R_2_CHA_2_DS_2_-VA and 0.70 and 0.846 for CHA_2_DS_2_-VA score respectively.

**Conclusions:**

R_2_CHA_2_DS_2_-VA score was found to be at least as good as CHA_2_DS_2_-VA score for predicting thromboembolism in Thai patients with NVAF. Similar to CHA_2_DS_2_-VA score, thromboembolism increased with incremental increase in R_2_CHA_2_DS_2_-VA score.

## Background

Ischemic stroke is a devastating complication in people with non-valvular atrial fibrillation (NVAF), and oral anticoagulants (OACs) have been proven effective for preventing stroke in these patients [1]. Recent clinical practice guidelines recommend that OAC should be prescribed in patients with a CHA_2_DS_2_-VA score of 1 or more (1 or more in male patients, and 2 or more in female patients) [2–4]. However, there are other stroke risks that are not included in this scoring system, such as renal disease. Renal dysfunction can contribute to change hemostatic systems such as increased pro-thrombotic blood components [5]. Although several trials reported renal dysfunction to be a predictor of thromboembolism in NVAF patients [6, 7], the Loire Valley Atrial Fibrillation Project revealed that renal impairment did not significantly improve the predictive value of the CHADS_2_ and CHA_2_DS_2_-VASc scores [8]. Moreover, data from a Chinese database was used to investigate the cutoff age for thromboembolic prediction. Previous studies found that age within the range of 50–64 years could enhance stroke risk stratification when added as a risk parameter to the CHA_2_DS_2_-VASc score [9–11]. Those data also revealed that the age threshold for increased stroke risk may be lower in Asians than in Caucasians [9–11]. However, there is no data specific to whether renal dysfunction and age within the range of 50–64 years added to CHA_2_DS_2_-VA score, which is known as R_2_CHA_2_DS_2_-VA score, can predict thromboembolism in NVAF patients. Previous population-based cohort study has shown that comparable stroke risk between women and men by using a nested case–control approach for analysis where women and men were matched on age and other confounding factors in time-dependent manner [12]. There has been a propose that female is a risk modifier rather than a risk factor for stroke in NVAF and CHA_2_DS_2-_VA should be used instead of CHA_2_DS_2-_VASc score [13]. The same group also reported a note of caution for the use of CHA_2_DS_2-_VA [14]. Accordingly, the aim of this study was (1) to compare the R_2_CHA_2_DS_2_-VA to CHA_2_DS_2_-VA score for predicting thromboembolism in Thai NVAF patients, and (2) to determine a sensitivity analysis of comparison with the conventional CHA_2_DS_2-_VASc score.

## Methods

Thai NVAF patients were prospectively enrolled in a nationwide multicenter registry from 27 hospitals in Thailand during 2014–2020. The COhort of antithrombotic use and Optimal INR Level in patients with non-valvular atrial fibrillation in Thailand (COOL-AF Thailand) registry is the largest NVAF registry in Thailand. The study protocol was approved by the institutional review boards (IRBs) of the Thailand Ministry of Public Health and of each participating hospital. Written informed consent was obtained by all participating patients, and all methods was conducted in accordance with the principles set forth in the Declaration of Helsinki and the International Conference on Harmonization for Good Clinical Practice Guidelines.

NVAF patients aged 18 years or more were recruited. Patients with prosthetic heart valve, rheumatic mitral valve disease, recent ischemic stroke within 3 months, NVAF from transient reversible cause, life expectancy less than 3 years, pregnancy, thrombocytopenia (< 100,000/mm^3^), myeloproliferative diseases, refusal to be enrolled, and/or could not come for follow-up were excluded.

Baseline demographic and clinical data of NVAF patients taking or not taking OACs were collected and recorded. Patient data were recorded on a case record form and in a centralized web-based system. The choice of OAC was determined at the discretion of each attending physician. The following data were collected: age, sex, baseline medical history, component parameters of R_2_CHA_2_DS_2_-VA and CHA_2_DS_2_-VA score, and type of antithrombotic medication. Patient data were recorded at follow-up visits scheduled for every 6 months. Any event outcomes that occurred during the preceding 6-month period, including death, non-fatal ischemic stroke or transient ischemic attack (TIA), or systemic embolism, were collected and recorded.

Each component of the CHA_2_DS_2_-VASc score was scored and recorded as C = congestive heart failure (1 point); H = hypertension (1 point); A = age ≥ 75 years (2 points); D = diabetes mellitus (1 point); S = stroke or TIA (2 points); V = vascular disease (1 point); A = age 65–74 years (1 point); and Sc = female sex (1 point). The R_2_CHA_2_DS_2_-VA score was defined as the CHA_2_DS_2_-VASc score including both R = renal dysfunction or estimated glomerular filtration rate (eGFR) ≤ 60 ml/min/1.73 m^2^ according to Chronic Kidney Disease Epidemiology Collaboration (CKD-EPI) formula [15] or renal replacement therapy (2 points) and A = age 50–74 years (1 point), but excluding female sex [12].

The main outcomes were thromboembolism, including ischemic stroke, TIA, and/or systemic embolism. Ischemic stroke was defined as a sudden onset of neurological deficit that lasted at least 24 h, but with no evidence of intracranial bleeding by computed tomography (CT) or magnetic resonance imaging (MRI) of brain [16]. TIA was defined as a sudden onset of neurological deficit that lasted less than 24 h [16]. Systemic embolism was defined as disruption of blood flow to other arteries, such as acute limb arterial occlusion or acute mesenteric arterial occlusion [17].

### Statistical analysis

The categorical data are described as number and percentage, and the continuous data are given as mean ± standard deviation (SD). The annual incidence rate of thromboembolism among patients in each R_2_CHA_2_DS_2_-VA and CHA_2_DS_2_-VA score category is demonstrated as rate per 100 person-years. Cox proportional hazards model was used to compare the rate of thromboembolism among patients in each risk score category with those with a risk score of 0. The results of that analysis are shown as hazard ratio (HR) and 95% confidence interval (CI). Receiver-operating characteristic (ROC) curve analysis was used to analyze the discrimination performance of R_2_CHA_2_DS_2_-VA and CHA_2_DS_2_-VA scores, and the results are shown as c-statistics [18]. Net Reclassification Index (NRI) and Integrated Discrimination Improvement (IDI) was performed based on the methods proposed in the previous publication [18] to determine the influence of R_2_CHA_2_DS_2_-VA on the reclassification of the study population. Calibration plot [19] was performed to determine the relation of predicted and observed probability between each scoring system and the observed events. We also performed sensitivity analysis by comparing R_2_CHA_2_DS_2_-VA with original CHA_2_DS_2_-VASc score. A *p*-value < 0.05 was considered statistically significant. All analyses were performed using SPSS statistical software version 18.0 (SPSS, Inc., Chicago, IL, USA) and R version 3.6.3 (www.r-project.org). NRI was performed by grouping study population by old and new model into 4 groups based on predicted probability of thromboembolism using contingency table. Kaplan–Meier (KM) estimate was then calculated from SPSS program. Calculation of number of case and control in each cell of the contingency table with KM estimate times person included. Calculation of Reclassification improvement in cases and Reclassification worsen in controls was performed and NRI was calculated. Calibration plot and IDI was performed by program R.

## Results

**Fig. 1:**
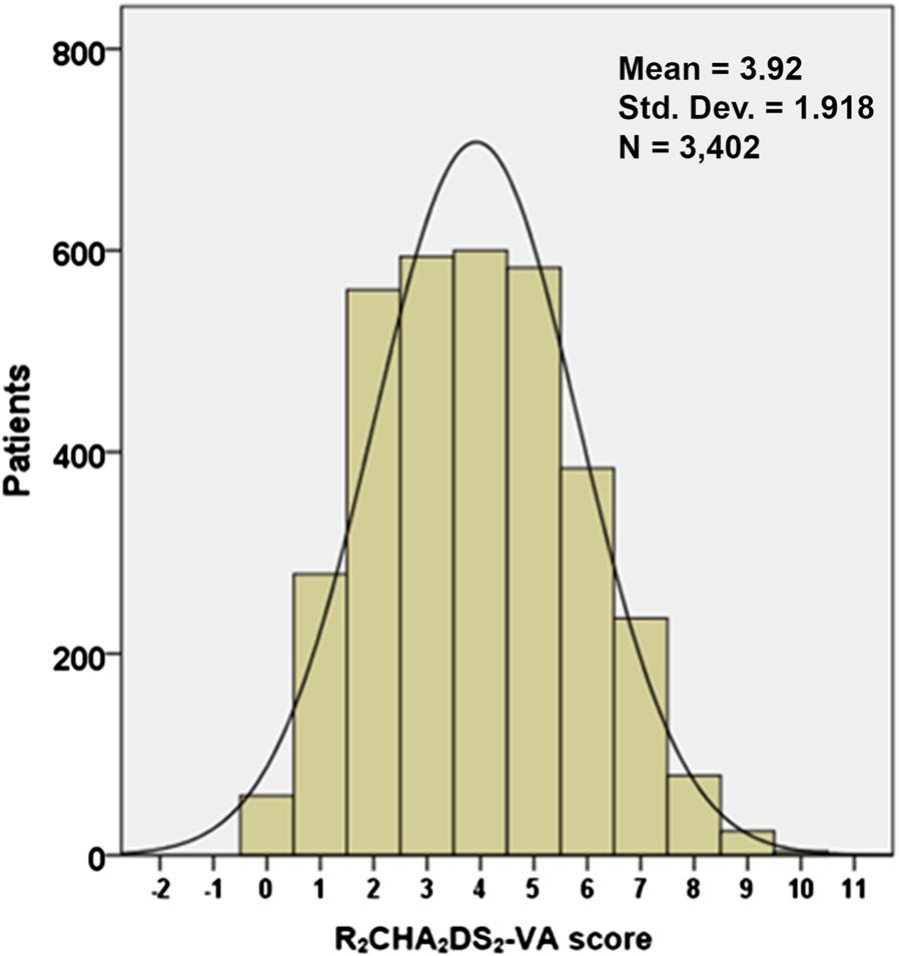
The distribution of patients according to R_2_CHA_2_DS_2_-VA score

A total of 3402 patients were enrolled in the COOL-AF Thailand registry during 2014–2020. The average age of patients was 67.38 ± 11.27 years, and 58.2% were male. Among all included patients, 46.9% had renal disease, 30.7% had a history of heart failure, and 17.1% had previous stroke or TIA. The average R_2_CHA_2_DS_2_-VA and CHA_2_DS_2_-VA scores were 3.92 ± 1.92 and 2.98 ± 1.43, respectively. Among all patients, 26.2% were prescribed antiplatelet, and 75.4% were prescribed OACs. The baseline characteristics of patients are shown in Table 1. The distribution of patients according to R_2_CHA_2_DS_2_-VA score is shown in Fig. 1.

**Table 1:** Baseline characteristics of NVAF patients compared between those on and not on OACs

Characteristics	Patients without OAC (n = 836)	Patients with OAC (n = 2566)	Total patients (n = 3402)
Age (years)	64.32 ± 12.39	68.37 ± 10.70	67.38 ± 11.27
Male sex	528 (63.2%)	1452 (56.6%)	1980 (58.2%)
R_2_CHA_2_DS_2_-VA score components			
Renal disease	335 (40.1%)	1259 (49.1%)	1594 (46.9%)
History of heart failure	235 (28.1%)	810 (31.6%)	1045 (30.7%)
Hypertension	467 (55.9%)	1861 (72.5%)	2328 (68.4%)
Age ≥ 75 years	180 (21.5%)	799 (31.1%)	979 (28.8%)
Diabetes mellitus	149 (17.8%)	690 (26.9%)	839 (24.7%)
Previous stroke or TIA	54 (6.5%)	538 (21.0%)	592 (17.4%)
Vascular disease	140 (16.7%)	441 (17.2%)	581 (17.1%)
Age 50–74 years	562 (67.2%)	1650 (64.3%)	2212 (65.0%)
Antithrombotic medications			
Antiplatelet	582 (69.6%)	308 (12.0%)	890 (26.2%)
Aspirin	521 (62.3%)	263 (10.2%)	784 (23.0%)
P2Y_12_ inhibitors	119 (14.2%)	81 (3.2%)	200 (5.9%)
Anticoagulant			
Warfarin	0 (0.0%)	2338 (91.1%)	2338 (68.7%)
Direct thrombin inhibitor	0 (0.0%)	82 (3.2%)	82 (2.4%)
Factor Xa inhibitors	0 (0.0%)	145 (5.7%)	145 (4.3%)
R_2_CHA_2_DS_2_-VA score			
0	43 (5.1%)	16 (0.6%)	59 (1.7%)
1	145 (17.3%)	134 (5.2%)	279 (8.2%)
2	150 (17.9%)	411 (16.0%)	561 (16.5%)
3	161 (19.3%)	433 (16.9%)	594 (17.5%)
4	114 (13.6%)	486 (18.9%)	600 (17.6%)
5	103 (12.3%)	480 (18.7%)	583 (17.1%)
6	69 (8.3%)	315 (12.3%)	384 (11.3%)
7	36 (4.3%)	199 (7.8%)	235 (6.9%)
8	10 (1.2%)	69 (2.7%)	79 (2.3%)
9	4 (0.5%)	20 (0.8%)	24 (0.7%)
10	1 (0.1%)	3 (0.1%)	4 (0.1%)
CHA_2_DS_2_-VA score			
0	48 (5.7%)	21 (0.8%)	69 (2.0%)
1	205 (24.5%)	197 (7.7%)	402 (11.8%)
2	223 (26.7%)	661 (25.8%)	884 (26.0%)
3	183 (21.9%)	748 (29.2%)	931 (27.4%)
4	95 (11.4%)	510 (19.9%)	605 (17.8%)
5	57 (6.8%)	290 (11.3%)	347 (10.2%)
6	20 (2.4%)	104 (4.1%)	124 (3.6%)
7	4 (0.5%)	31 (1.2%)	35 (1.0%)
8	1 (0.1%)	4 (0.2%)	5 (0.1%)
R_2_CHA_2_DS_2_-VA score	3.22 ± 1.97	4.15 ± 1.84	3.92 ± 1.92
CHA_2_DS_2_-VA score	2.42 ± 1.46	3.17 ± 1.63	2.98 ± 1.43

Data shown as mean ± standard deviation or number and percentage

NVAF, non-valvular atrial fibrillation; SD, standard deviation; OACs, oral anticoagulants; TIA, transient ischemic attack

**Fig. 2:**
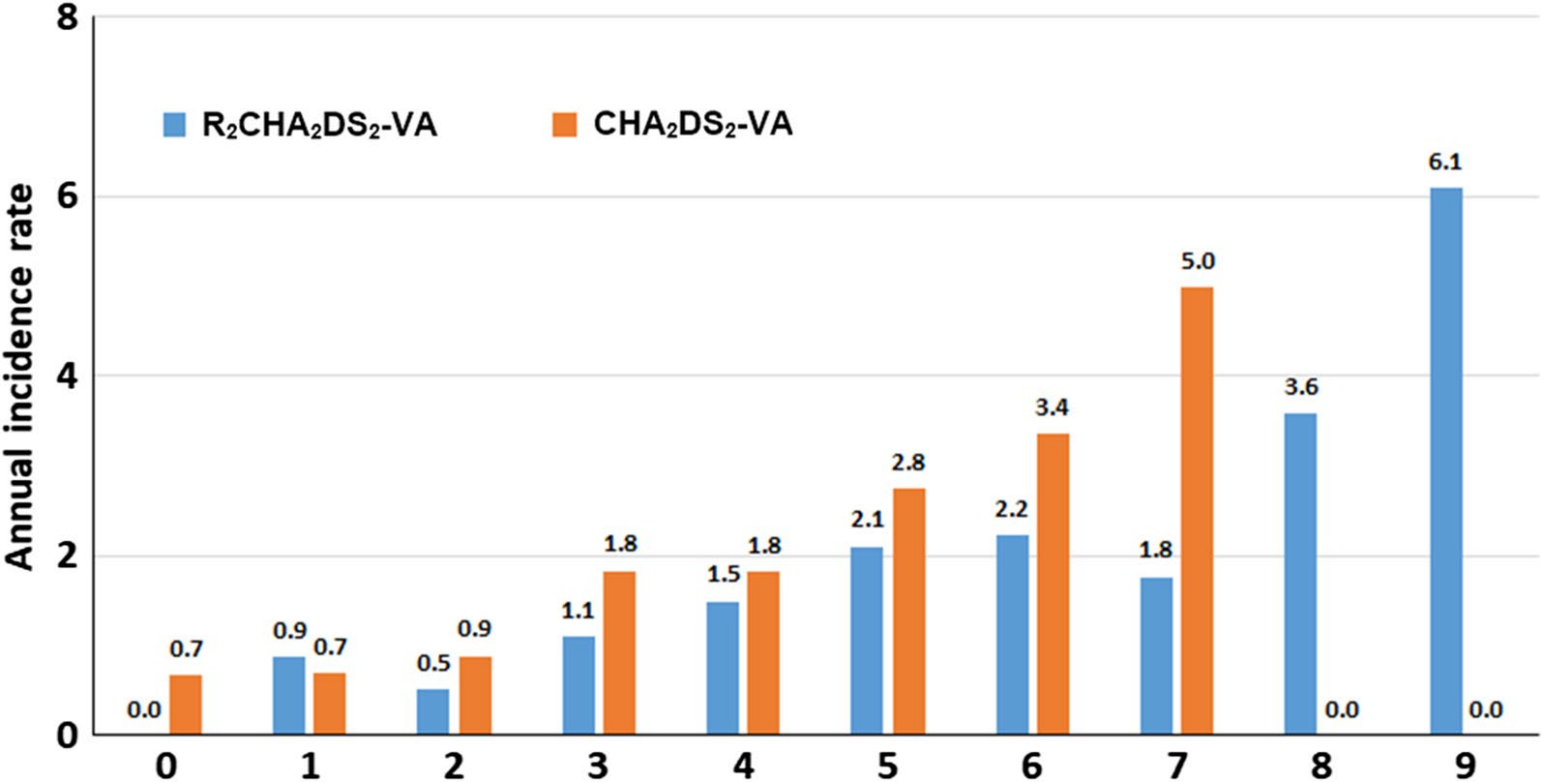
Cumulative annual incidence of thromboembolism compared between R_2_CHA_2_DS_2_-VA score and CHA_2_DS_2_-VA score

Annual thromboembolic risk increased with incremental increase in R_2_CHA_2_DS_2_-VA and CHA_2_DS_2_-VA scores (Table 2 and Fig. 2). OACs is shown to have beneficial effect in the protection of ischemic stroke/TIA for those with NVAF patients with R_2_CHA_2_DS_2_-VA ≥ 2 (adjusted HR: 0.630, 95% CI 0.413–0.962) and a trend toward a protective effect for those with the score of 1 or more (adjusted HR: 0.726, 95% CI 0.483–1.090, *p* = 0.122) (Table 3).

**Table 2:** Annual thromboembolic risk in patients stratified by R_2_CHA_2_DS_2_-VA score and non-sex CHA_2_DS_2_-VASc score

Risk scoring system	Number of thromboembolisms	Annual incidence rate (per 100 person-years)	p-value for trend
R_2_CHA_2_DS_2_-VA score			< 0.001
0	1	0.78	
1	5	0.87	
2	6	0.51	
3	14	1.10	
4	19	1.50	
5	26	2.11	
6	18	2.24	
7	9	1.77	
8	6	3.58	
9	3	6.09	
Total	107	1.49	
CHA_2_DS_2_-VA score			< 0.001
0	1	0.68	
1	7	0.85	
2	15	0.81	
3	30	1.50	
4	25	1.96	
5	17	2.30	
6	9	3.37	
7	3	4.06	

HR, hazard ratio; 95% CI, 95% confidence interval

^*^HR of risk of thromboembolism in patients in each risk score category compared to patients with a risk score of 0

**Table 3:** Risk of thromboembolism

Antithrombotic strategy	Thromboembolism		
	Annual incidence rate	Adjusted HR (95% CI)	*p*-value
R_2_CHA_2_DS_2_-VA score ≥ 1			
No anticoagulant	1.86	–	–
Anticoagulant	1.36	0.726 (0.483–1.090)	0.122
R_2_CHA_2_DS_2_-VA score ≥ 2			
No anticoagulant	2.16	–	–
Anticoagulant	1.38	0.630 (0.413–0.962)	0.032

A *p*-value < 0.05 indicates statistical significance

HR, hazard ratio; 95% CI, 95% confidence interval

**Fig. 3:**
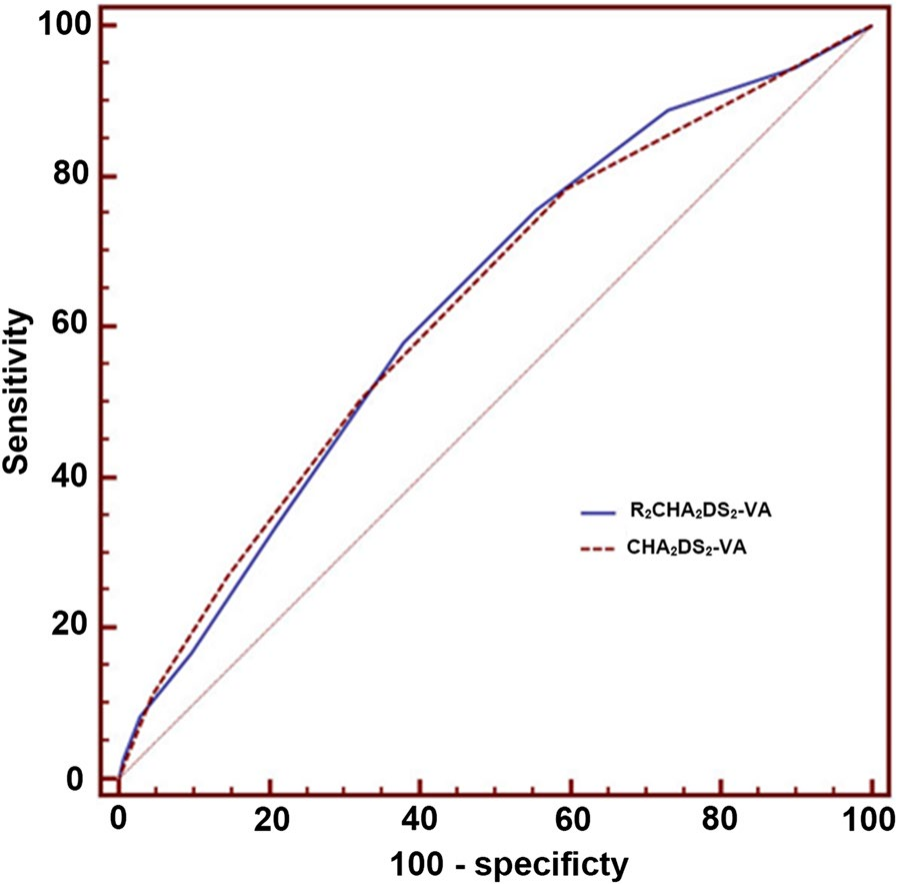
Receiver operating characteristic (ROC) curve comparing c-statistics between R_2_CHA_2_DS_2_-VA score and CHA_2_DS_2_-VA score (*AUC* area under the curve)

The discrimination performance of R_2_CHA_2_DS_2_-VA and CHA_2_DS_2_-VA risk scores are shown as c-statistic values of 0.630 (95% CI 0.61–0.65) and 0.627 (95% CI 0.61–0.64), respectively (Fig. 3).

## Net reclassification index (NRI)

Calculation of predicted probability for 3-year risk of ischemic stroke/TIA was performed using Cox proportional Hazard model of all factors of each scoring system. Afterward, we classified patients into 4 risk groups as follows: 0–2%, 2–4%, 4–6%, and ≥ 6% risk of ischemic stroke/TIA based on the predicted probability. From Kaplan–Meier (KM) estimate, we calculated the number of cases that move to higher or lower risk groups with the use of R_2_CHA_2_DS_2_-VA as compared to the risk groups classified by CHA_2_DS_2_-VA score. We found that 4.7% of cases was moved to a higher risk group and 4.8% of controls was moved to a lower risk group. The NRI and IDI were 2.2% and 0.02% indicating that CHA_2_DS_2_-VA score performed slightly better than CHA_2_DS_2_-VA score in predicting ischemic stroke/TIA. For patients who were on OAC which was the majority of patients, the NRI was 4.32% for R_2_CHA_2_DS_2_-VA as compared to CHA_2_DS_2_-VA score.

## Calibration plot

**Fig. 4:**
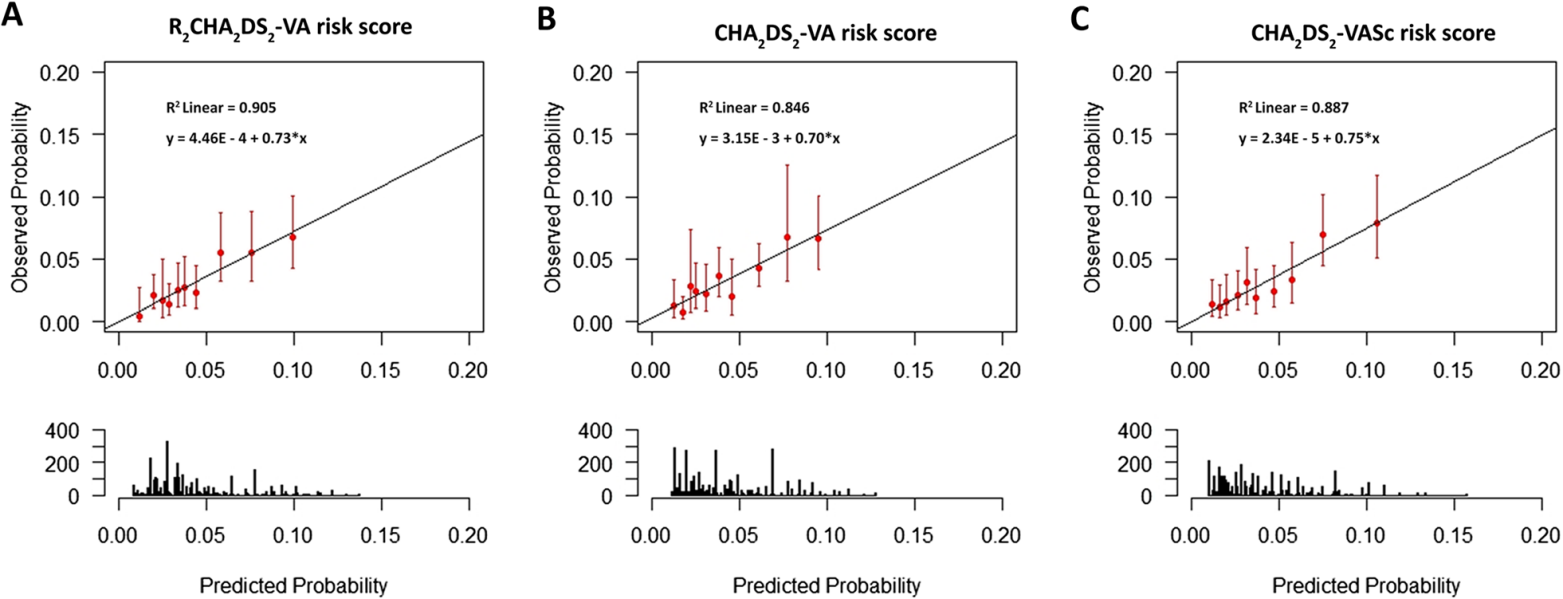
Calibration plot for 10 equal groups of predicted probability with predicted probability of event based on R_2_CHA_2_DS_2_-VA (**A**), CHA_2_DS_2_-VA score (**B**), and original CHA_2_DS_2_-VASc score (**C**) on X-axis and observed event on Y-axis

Predictive model for ischemic stroke/TIA at 3 years was derived using the formula P_IS/TIA_ at 3 years = 1 − S0(t)^exp (Prognostic Index)^ where P = predicted probability, IS = ischemic stroke, TIA = transient ischemic attack, S0(t) = average survival probability at time, prognostic index is calculated from Cox proportional Hazard model using all factors of each scoring system). Calibration plot was performed for 10 equal groups of predicted probability with predicted probability of event based on R_2_CHA_2_DS_2_-VA and CHA_2_DS_2_-VA score on X-axis and observed event on Y-axis (Fig. 4A, B). Calibration plot of R_2_CHA_2_DS_2_-VA showed a slightly higher slope compared to CHA_2_DS_2_-VA score and the R2 which is an index of goodness-of-fit measure of the linear model was higher for R_2_CHA_2_DS_2_-VA compared to CHA_2_DS_2_-VA score. Calibration slope of R_2_CHA_2_DS_2_-VA, CHA_2_DS_2_-VA, and original CHA_2_DS_2_-VASc indicate a good agreement between predicted probability and observed outcomes among group of patients.

## Sensitivity analysis

We performed sensitivity analysis by comparing R_2_CHA_2_DS_2_-VA to the original CHA_2_DS_2_-VASc score. The calibration slope was similar for the 2 scoring systems. The R2 was slightly higher for R_2_CHA_2_DS_2_-VA compared to CHA_2_DS_2_-VASc score (Fig. 4C). NRI and IDI for R_2_CHA_2_DS_2_-VA compared to CHA_2_DS_2_-VASc score was 0.42% and 0.14% respectively. A total of 5.4% of cases moved to higher risk group and 9.1% of controls moved to a lower risk group.

## Discussion

Based on current knowledge, CHA_2_DS_2_-VASc score is recommended for stroke risk assessment according to standard clinical practice guidelines, and female sex is a risk modifier rather than a risk factor for ischemic stroke. However, some stroke risks, such as renal dysfunction and age 50–64 years, are not included in this scoring system. Renal dysfunction promotes thrombosis by an increase in platelet activity, activation of the renin–angiotensin–aldosterone system (RAAS) and alteration in blood vessel wall contractility due to inflammation resulting in prothrombotic state [15]. Previous Korean study reported the inclusion of chronic kidney disease (CKD) into the CHA_2_DS_2_-VASc score and deletion of sex, which resulted in the CHA_2_DS_2_VAK score [7]. This novel scoring system demonstrated improved ability to discriminate intermediate-risk patients. Additionally, a previous study from Hong Kong reported that NVAF patients aged 50 to 64 years had increased stroke risk despite having a low CHA_2_DS_2_-VASc score [10]. That study concluded that patients aged less than 50 years had a significantly lower risk of stroke.

Our study showed that the R_2_CHA_2_DS_2_-VA score can predict thromboembolic events in NVAF patients. An increased R_2_CHA_2_DS_2_-VA score led to more annual thromboembolic risk. Compared with non-anticoagulated patients, anticoagulated patients had a lower risk of thromboembolism with borderline statistical significance (adjusted HR: 0.726, 95% CI: 0.483–1.090, *p* = 0.122). This finding suggests that OACs may reduce thromboembolism in NVAF patients with a higher R_2_CHA_2_DS_2_-VA score. This scoring system included renal dysfunction and age 50–64 years into the CHA_2_DS_2_-VASc score, but female sex was removed. As a result, the R_2_CHA_2_DS_2_-VA score has more risk factor parameters than the CHA_2_DS_2_-VASc score. We demonstrated that R_2_CHA_2_DS_2_-VA and CHA_2_DS_2_-VA score had a similar c-statistic values, 0.630 (95% CI 0.61–0.65) and 0.627 (95% CI 0.61–0.64), for ischemic stroke/TIA. In the NRI analysis, we showed that R_2_CHA_2_DS_2_-VA had a slightly higher NRI compared to CHA_2_DS_2_-VA system. The R2 of calibration plot graph of predicted risk and observed risk of R_2_CHA_2_DS_2_-VA also slightly higher than CHA_2_DS_2_-VA score. We also had the results of the comparison of R_2_CHA_2_DS_2_-VA and the original CHA_2_DS_2_-VASc score which showed that R_2_CHA_2_DS_2_-VA was at least as good as the original CHA_2_DS_2_-VASc score. These results suggested that by adding renal function data and the inclusive of a lower age group might have an additional value or at least as good as CHA_2_DS_2_-VA and the original CHA_2_DS_2_-VASc score.

There are some possible explanations why the R_2_CHA_2_DS_2_-VA score did not demonstrate better discriminative performance than the CHA_2_DS_2_-VASc score despite having more risk factor parameters. First, the addition of renal dysfunction and age 50–64 years led to higher R_2_CHA_2_DS_2_-VA scores, while lower CHA_2_DS_2_-VA scores led to a comparable rate of thromboembolic events. It is also possible that giving two points for renal dysfunction may overestimate thromboembolic events in this setting because CKD had an HR of 1.62 for predicting thromboembolic events in Korean population [7]. Second, most NVAF patients (75.5%) in this study had been taking OACs while most patients in previous CHA_2_DS_2_VAK, modified CHA_2_DS_2_VASc and CHA_2_DS_2_VASc trials had no OACs [6, 8]. As shown in the results, the NRI of R_2_CHA_2_DS_2_-VA compared to CHA_2_DS_2_-VA score, was greater in patients who are on OAC. Therefore, the number of thromboembolic events was lower in our study when compared with previous non-anticoagulant NVAF trials, which explains the comparable discriminative performance between the two scoring systems.

### Strengths and limitations

This study also has some limitations. First, this study included both anticoagulated and non-anticoagulated NVAF patients, which resulted in a lower thromboembolic event rate than the rates reported in the previous non-anticoagulated risk score trials mentioned above. Nevertheless, the R_2_CHA_2_DS_2_-VA score had acceptable discriminative performance, with a c-statistic value of 0.630 compared with 0.606 in a previous trial [20]. Second, our study recruited only Thai NVAF patients, so our results may not be generalizable to other races. Despite these limitations, this study had some strengths. First, this study introduces the R_2_CHA_2_DS_2_-VA score which can predict thromboembolism in NVAF patients. This novel risk score included other stroke risks such as renal dysfunction and lower cutoff age for thromboembolic prediction in addition to CHA_2_DS_2_VASc score leading to consider anticoagulation in broader AF population especially patients with renal dysfunction or age of 50–64 years with CHA_2_DS_2_VASc of 0. Second, this is a multicenter nationwide study in Thailand. Lastly, the events in this study were adjudicated.

## Conclusions

R_2_CHA_2_DS_2_-VA score was found to be comparable to CHA_2_DS_2_-VA score for predicting thromboembolism in Thai patients with NVAF. Similar to CHA_2_DS_2_-VA score, thromboembolism increased with incremental increase in R_2_CHA_2_DS_2_-VA score.

## Data Availability

The dataset that was used to support the results and conclusion of this study are included within the manuscript. Additional data are available upon contacting Rungroj Krittayaphong at rungroj.kri@mahidol.ac.th with the reasonable request.

## Abbreviations

AF: Atrial fibrillation
CKD-EPI: Chronic Kidney Disease Epidemiology Collaboration
COOL-AF Thailand: COhort of antithrombotic use and Optimal INR Level in patients with non-valvular atrial fibrillation in Thailand
CI: Confidence interval
CT: Computed tomography
eGFR: Estimated glomerular filtration rate
HR: Hazard ratio
IRB: Institutional review board
MRI: Magnetic resonance imaging
NVAF: Non-valvular atrial fibrillation
OAC: Oral anticoagulant
ROC curve: Receiver operating characteristic curve
SD: Standard deviation
TIA: Transient ischemic attack
